# Body Composition in Fussy-Eating Children, with and without Neurodevelopmental Disorders, and Their Parents, Following a Taste Education Intervention

**DOI:** 10.3390/nu15122788

**Published:** 2023-06-18

**Authors:** Sigrun Thorsteinsdottir, Ragnar Bjarnason, Helga G. Eliasdottir, Anna S. Olafsdottir

**Affiliations:** 1 Faculty of Health Promotion, Sport and Leisure Studies, School of Education, University of Iceland, Stakkahlid, 105 Reykjavik, Iceland; helgag@gmail.com (H.G.E.); annaso@hi.is (A.S.O.); 2 Faculty of Medicine, School of Health Sciences, University of Iceland, Laeknagardur 4th Floor, Vatnsmyrarvegur 16, 101 Reykjavik, Iceland; ragnarb@landspitali.is; 3 Department of Pediatrics, National University Hospital, Hringbraut, 101 Reykjavik, Iceland

**Keywords:** BMI, standard deviation scores, fat percentage, body composition measurements, bioelectrical impedance analysis, neurodevelopmental disorders, fussy eating, autism spectrum disorder, ADHD, parent–child dyads

## Abstract

Fussy eaters may have an increased risk of becoming overweight or obese as adolescents, with fussy eating and weight status also correlating with neurodevelopmental disorders (NDs) such as autism spectrum disorder (ASD) and attention deficit/hyperactivity disorder (ADHD). Further, maternal and children’s weight status relationships are well-established. In this study, we analyzed the body composition of parent–child dyads using bioelectrical impedance analysis (BIA). Fifty-one children aged 8–12 years, with an ND (*n* = 18) and without (*n* = 33), and their parents, participated in a 7-week food-based Taste Education intervention with 6-month follow-up. The paired *t*-test was used to compare differences in body composition based on children’s ND status. In logistic regression analysis, odds of children being in the overweight/obese or overfat/obese categories increased by a factor of 9.1 and 10.6, respectively, when having NDs, adjusting for parents’ BMI (body mass index) or fat percentage (FAT%). Children with NDs and their parents had significantly higher mean BMI-SDS (BMI standard deviation score) and FAT% at pre-intervention than children without NDs and their parents. Mean BMI-SDS and FAT% lowered significantly between time points for children with NDs and their parents but not for children without NDs or their parents. The findings underline the need for additional exploration into the relationships between children’s and parents’ body composition based on children’s ND status.

## 1. Introduction

In recent decades, the prevalence of overweight and obesity in children and adults has increased considerably worldwide [1,2,3,4]. Obesity is considered a chronic, relapsing, and progressive disease, affecting individuals in all environments and demographics, particularly those in the lower socioeconomic brackets [5,6,7,8]. 

The causes of obesity are multifaceted and complex and include environmental, physiological, genetic, and behavioral factors [8,9,10]. There is also evidence of parental feeding practices and obesity being tied to children’s eating psychopathology and weight trajectories [11]. In one study, mothers who were overweight or had obesity were involved in less healthy feeding practices with their children than mothers in the healthy weight range [12]. Similar reports have been published on the positive correlations between parents’ and children’s weight status [13,14,15]. Other mechanisms affecting children’s weight status may also be related to their eating behaviors, such as fussy eating and food neophobia, in which a limited diet may affect children’s general health and growth [16,17,18]. Fussy eating is often seen in children with obesity [17,19,20], as fussy eaters may be at a higher risk for becoming overweight or have obesity as older children and adolescents, possibly due to a penchant for calorie-dense foods and sweet beverages, and rejection of fruits and vegetables [21]. However, some researchers have not been able to determine the association between children’s increased weight status and fussy eating [22], with some studies even reporting the risk of underweight in fussy-eating children [23,24,25,26,27,28], especially in those who are not typically developing (TD). 

### 1.1. Fussy Eating and Neurodevelopmental Disorders

Numerous interpretations exist regarding problematic eating behaviors, encompassing food neophobia (disinclination toward unfamiliar or unknown foods), fussy or selective eating (refusing a substantial portion of both new and familiar foods), exhibiting displeasure during mealtimes, and engaging in oppositional behaviors or tantrums [29,30,31]. Typically, fussy eating results in a limited diet, characterized by a notably low variety in consumed foods [16,32,33]. Given that fussy eating encompasses food neophobia, both terms are often collectively referred to as fussy eating.

Fussy eating and other selective eating behaviors in children are strongly correlated with neurodevelopmental disorders (NDs) such as autism spectrum disorder (ASD) and attention deficit/hyperactivity disorder (ADHD), as NDs may severely affect eating behaviors [34,35,36,37,38]. Significantly, NDs may be a mediating factor in children’s weight status and fussy-eating behaviors [31,39,40], in which children with NDs, especially those with ASD rather than ADHD, may be hypersensitive to food textures, smells, and taste [38,41,42,43,44]. Sensory sensitivities may limit children’s food variety, and although fussy eating often peaks between 2 and 6 years old, those with the most difficulties in accepting new foods and different sensory aspects of food will not grow easily out of their fussy eating [44]. Parents of fussy eaters, with and without NDs, frequently report their children refusing fruit and vegetables, whole grains seeds, and legumes, which may cause parental concern about the child’s well-being [45,46,47,48] and weight increase as well as decrease [25,49,50]. In addition, parental worrying, and pressure to eat may cause stress in children, and exacerbate mealtime behavior problems, thus reducing mealtime enjoyment and food variety [31,46,51,52,53,54,55,56].

Despite ASD often receiving the stronger focus on challenging eating behaviors in children with NDs, a relatively recent study pointed to fussy eating being similar across NDs, where children with ADHD had similar problems to those with ASD [57]. Overconsuming calorie-rich food, sweets, and sugary drinks is common in ADHD [58,59,60,61]. This unhealthy eating behavior may translate into disordered eating, including binge-eating disorder, and higher BMI among men and women, including overweight and obesity [62,63,64,65]. The risk for disordered eating is especially high when ADHD symptoms are not treated with support measures or medication [59,63]. The prevalence of fussy eating is considerably higher in children with NDs than in TD children, impacting close to 80% of children with ASD [38,41,66] and up to 40% in children with ADHD [38,67]. 

### 1.2. Weight Status of Icelandic Children and Adults

During the past decades, childhood obesity has increased in Iceland, as it has globally [4,68,69,70,71,72,73]. The 2022 report from The Development Centre for Primary Healthcare in Iceland (DCPHI) stated there was 7.0% overall obesity in children aged 6, 9, 12, and 14 (*n* = 17,899), reporting data from all primary/elementary schools in Iceland. The rate is up from 6.5% (*n* = 17,533) in 2019–2020. Less than 1.3% of boys and girls in all age groups are reported as being underweight [72,73], with approximately 74% of children registered in the healthy weight range and 18% as overweight [72]. Icelandic children have some of the highest prevalence of obesity in Europe [74], corresponding with trends in Icelandic adults; according to a recent OECD survey (Organisation for Economic Co-operation and Development), Iceland now ranks highest in Europe for obesity in adults (27%) followed closely by Malta and Lithuania (both 26%), and the UK (21%) [75]. 

### 1.3. Body Composition Measurements

The BMI (body mass index, kg/m^2^), adjusting for age and sex to categorize weight status in children, is one of the most widely used health indicators, despite its limitations [76,77]. For example, the BMI does not differentiate between fat-free mass (FFM) and fat mass [78], which makes measures of weight and height alone poor indicators of health, or as an index of adiposity, as substantial fat mass may lead to excessive fat accumulation in obesity [79,80]. Further, the BMI does not adjust for ethnic backgrounds, activity levels, or females’ menarche status, which may affect body composition [79,81,82,83,84,85,86]. 

Body composition measurements in children are inherently challenging due to the precipitous growth-related changes in, for example, weight, height, fat mass, and FFM. Still, they are considered essential in clinical care [87]. Anthropomorphic measures are typically used as markers in the relationship between obesity and cardiovascular disease [81] and indicators of a heightened risk of several chronic diseases [69,88]. For example, rapid growth rates, especially during teenage years, may raise cancer risk later in life [89]. Further, chronic or cardiovascular disease indicators may include waist-to-height ratio, skinfold thickness, hip and waist circumference, and BMI [81]. 

Several body composition measurement techniques are available, such as dual-energy X-ray absorptiometry (DEXA), magnetic resonance imaging (MRI), and bioelectrical impedance analysis (BIA) [79,90]. Of the various techniques, BIA is considered the least invasive, quickest, safest, and one of the most reliable and cost-effective techniques, reproducible with <1% error on repeated measurements [90]. Furthermore, for its ease of use, it is also especially suitable for children. This technique is indicated for use for healthy children 5–17 years old and adults with active, moderately active, to inactive lifestyles [79,83,91,92,93,94]. The Tanita Body Composition Analyzer—Tanita MC-780 [83] is indicated for use in the measurement of weight and impedance and the estimation of BMI, total body fat percentage (FAT%) and segmental fat percentage, weight (fat mass, kg), bone mass (kg), and fat-free mass (FFM, kg). FAT% signifies the proportion of fat to the total body weight. Fat mass denotes the actual weight of fat within the body. FFM comprises tissue, bone, muscle, and water in the body [83,90]. The calculation of different components through BIA presumes that 73% of FFM is body water, a good electrical conductor, while fat mass is a poor conductor due to its lower hydration [94]. 

### 1.4. Relationships between Parents’ and Children’s Body Composition

Although the relationships between parents’, especially maternal, and children’s weight status are well-established [11,12,95,96,97], to the author’s best knowledge, no study exists on the body composition of parents whose children have fussy eating, with or without NDs. 

A deeper understanding of the body composition of children with and without NDs and the associations with their parents’ body composition is essential, as deviations from a healthy status may be indicative of issues requiring further attention, such as poor nutrient profiles or stringent food choices. Problems affecting children’s eating behaviors may, in the long term, increase weight-related difficulties for children and their parents, including decreased or increased weight in children [22] and reduced food variety [17,51], as well as increased parental stress and more negative mealtime experiences [98,99,100,101]. The factors underlying problematic eating behaviors may, individually or collectively, impact the weight trajectory of both parents and children [102]. In this study, we analyzed the body composition (BMI, BMI-SDS, and FAT% using BIA) of parents and children, with and without NDs, participating in a food-based intervention. The purpose of this study was to gain insight into differences and associations between parents’ and fussy-eating children’s body composition, based on ND status, i.e., based on children with or without neurodevelopmental disorders. To the best of our knowledge, body composition in a similar sample has not been investigated previously or in other food-based interventions. 

## 2. Materials and Methods

The data used in this study were cross-sectional, comparing participants at pre-intervention and six months follow-up, based on a longitudinal, randomized controlled study of a Taste Education intervention. Parent–child dyads who completed both measurements were used in the study. For more detailed information on the original study’s methods, see Thorsteinsdottir, Njardvik et al. [103]. 

### 2.1. Measures

Demographic information, including children’s age, sex, and medication use, and parental education, occupation, and marital status, was obtained from the screening questionnaires developed by the researchers [103].

#### 2.1.1. Anthropometric Measures

Height was measured to the nearest 0.1 cm using a portable stadiometer (Stadiometer, Ulm, Germany). Weight was measured to the closest tenth of a kilogram using an electronic weighing scale (Tanita MC-780 MA Multi Frequency Segmental Body Composition Analyzer) [83]. The children wore light clothing and were instructed to remove shoes and socks and empty all pockets before stepping on the scale. A correction for clothing weight was subtracted for participants: −1.0 kg for parents and −0.5 kg for children. Weight status (underweight, healthy weight, overweight, and obesity) was defined according to the International Task Force of Obesity [104]. BMI-SDS (body mass index standard deviation scores) were derived from BMI reference values for Swedish children adjusting for age and sex [105]. Participants were measured with an empty bladder, by the same team members, at the same time of day, and wearing similar clothes for both measurements. The team members had extensive experience measuring children and adults using the Tanita MC-780. 

#### 2.1.2. Measure of Fussy Eating

Children’s fussy eating was evaluated by a parent-report measure, the Children’s Eating Behavior Questionnaire (CEBQ) [106], containing 35 items each, rated on a five-point Likert scale ranging from ‘never’ to ‘always’. CEBQ comprises eight scales: Food fussiness, Slowness in eating, Food responsiveness, Emotional over-eating, Emotional under-eating, Enjoyment of food, Desire to drink and Satiety responsiveness. The psychometric properties of the instrument have been previously reported [106,107]. Further, the psychometric properties of the Icelandic version [108] have suggested good correspondence to Wardle and colleagues’ original research [106]. An average of the items for each subscale was calculated. A high score on food fussiness indicates high levels of fussy eating. A high score for enjoyment of food suggests high levels of enjoyment. Both subscales were the focus of the current study. Internal reliability coefficients (Cronbach’s alpha) for all subscales ranged from good (0.71) for satiety responsiveness to very good for emotional over-eating (0.86). In this present study, Cronbach’s alpha was very good for food fussiness (0.85) and enjoyment of food (0.85).

**Figure 1:**
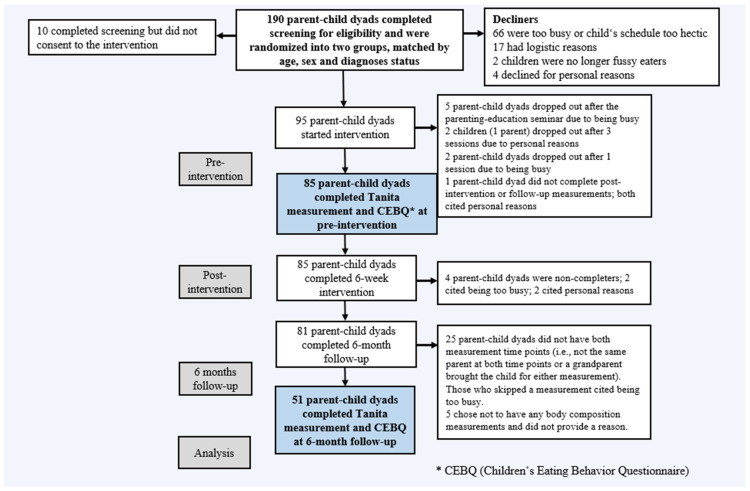
Flow diagram of intervention by stages of the study showing Tanita body composition and CEBQ (Children’s Eating Behavior Questionnaire) measure points in blue.

Parents answered questions regarding the CEBQ at the start of the intervention, with follow-up at six months (Figure 1). Questionnaires were administered and stored online using Qualtrics software (Qualtrics, Provo, UT, USA).

#### 2.1.3. Tanita Body Composition Analyzer

For analysis, the patient’s sex, date of birth, and height were entered into the Tanita software. The ‘standard’ body type was selected for all participants as none were professional athletes, or training in heavy weightlifting or bodybuilding. All participants were asked to stand on the device platform and to keep their hands by their sides while gripping the machine’s handles. Each measurement took less than one minute. 

### 2.2. Participants

The participants in this study were children aged between 8–12 years old and their parents. To ensure that children’s ADHD and ASD diagnoses were valid, all were required to have been diagnosed by one of the three main Icelandic diagnostic centers, which all utilize standardized diagnostic protocols and instruments. Of the 81 parent–child dyads that completed the Taste Education intervention [103], 51 completed two body composition analysis measurements, at baseline and six months follow-up (Table 1). Twenty-five parent–child dyads did not have both measurement time points (i.e., not the same parent at both time points or a grandparent brought the child for either measurement). Those who skipped a measurement cited being too busy. Five chose not to have any body composition measurements and did not provide a reason (Figure 1). There were no significant differences in any of the background measures for parents or children between the 51 participants who received both measurements and the 30 that had either one measurement or chose not to have any. 

The majority of parents were mothers, and all participants were Icelandic and spoke Icelandic. The participants in the study were invited via email lists in collaboration with the Icelandic ASD and ADHD societies and through advertisements published on a website set up expressly for the study. All children attended mainstream schooling. Inclusion criteria encompassed Icelandic-speaking participants and fussy-eating children 8–12 years of age [103]. Descriptive characteristics of the participants at pre-intervention are provided in Table 1.

### 2.3. Procedures

Parents volunteered to partake in the study and were invited to answer a screening questionnaire online about themselves and their children for consent and eligibility. Participants were not financially rewarded for participating in the study, although the children received a graduation certification and were gifted a tote bag for completing the Taste Education program. The sessions took place at a home economics teaching kitchen, within the School of Education at the University of Iceland, from 2018 to 2019. The Taste Education program was tailored as an after-school program being delivered midweek, at the same time of day for all participants. The intervention was divided into two components, i.e., (a) two parenting-education sessions, two hours each, prior to the first session with the children, and (b) six kitchen sessions, 90 min each, comprising food preparation skills and games as a base for sensory and taste education in the teaching kitchen with parent–child dyads (for further details of the kitchen sessions see Thorsteinsdottir et al. [103]). The sensory-based education centred on food-themed games, introducing texture-based exercises, sounds of food in the mouth, and cooking and baking simple recipes (re-introducing the now familiar ingredients). Each session built upon the previous one with food items used previously, re-introduced in the following session. Exercises were based on repeated exposures (repetition of seeing foods, handling them, and tasting). For details on the foods introduced in the Taste Education intervention, and changes in acceptance, see Thorsteinsdottir et al. [103].

### 2.4. Statistical Analyzes 

Data were analyzed using R Studio 4.2.2 [109]. Descriptive statistics were calculated and are presented as *n* (%) and mean (SD). Cases with missing data were excluded listwise, and only parent–child dyads with two body composition measurements were included in the analyses. 

Parents‘ BMI status was divided into four groups, adjusting for age and sex: underweight, healthy weight, overweight, and obesity [110]. Likewise, children were classified into four categories based on their BMI status adjusting for age and sex: underweight (less than the 5th percentile), healthy weight (5th percentile to <85th percentile), overweight (85th percentile to <95th percentile), and obesity (95th percentile or greater) [104]. 

Body fat percentages were based on body fat ranges, with cut-offs to define underfat, normal, overfat, and obesity, adjusting for age and sex set at the 2nd, 85th, and 95th centiles for children [111]. Parents’ body fat percentage was also divided into four groups, adjusting for age and sex [83,112].

The paired t-test was used to compare differences in Tanita body composition measurements based on children’s ND status and between parents based on their children’s ND status. To retain statistical power, categories for BMI and FAT% were collapsed into binary variables; BMI: underweight/healthy weight vs. overweight/obese (underweight/healthy < 18.5–24.99, adjusted for age and sex) [110] and FAT%: underfat/healthy vs. overfat/obese (underfat/healthy < 16.0–34.99%, adjusted for age and sex) [112].

A comparison between children’s and parents’ body composition is presented using boxplots, crosstabs, paired- and independent samples *t*-test, and the chi-square or Fisher’s exact test where applicable. Multivariable associations between children’s ND status and children’s BMI or FAT% status were assessed using binary logistic regression, adjusting for parents’ BMI or FAT% status. Estimated associations were described with odds ratios and 95% confidence intervals (CIs). *p*-values of <0.05 were considered statistically significant. Although multiple hypotheses tests were conducted, unadjusted alphas were reported [113,114]. 

## 3. Results

### 3.1. Sample Characteristics

The demographic characteristics of the 51 participants are presented in Table 1. More than a third of the children (35.3%) had an ND. The mean age of participating children was 10.0 (*SD* = 1.4; range 7–13 years), and 41.2% were female. Two of the 18 children with an ND were female (11.1%). The mean BMI-SDS for the children was 0.27 (*SD* = 1.21). The majority of parents were mothers (82.3%), 66.7% had a full-time occupation, and 72.5% had obtained a university education. The mean age of parents was 42.2 (*SD* = 4.9; range 31–52 years old), and the mean parental BMI was 26.9 (*SD* = 5.1). Two of the 18 parents whose children had an ND were fathers (11.1%). 

### 3.2. Body Compositions Based on Sex

Although not the focus of the present study, differences in body compositions based on sex were calculated before analyzing measures based on ND (Table 2). For children, the proportion of boys with an ND (88.9%) was significantly higher than for girls (*χ*^2^(1, *N* = 51) = 8.55, *p* = 0.003), so analyses for children’s measures were run separately before examining measures based on ND (Table 2). There were no significant differences between girls and boys based on age (*t*(34) = 1.286, *p* = 0.207), weight (*t*(49) = 0.975, *p* = 0.334), height (*t*(39) = 1.910, *p* = 0.063), BMI (*t*(48) = −0.055, *p* = 0.956), fat mass (*t*(45) = 0.162, *p* = 0.872), FAT% (*t*(49) = 0.253, *p* = 0.802), or FFM (*t*(48) = 1.485, *p* = 0.144). The only significant difference between girls and boys was for bone mass: boys had higher bone mass (*M* = 1.61 kg, *SD* = 0.36) than girls (*M* = 1.39 kg, *SD* = 0.31), (*t*(47) = 2.273, *p* = 0.028). 

Since the proportion of parents’ sex did not differ by their children’s ND status, (*χ*^2^(1, *N* = 51) = 0.27, *p* = 0.603), parents’ measures were run based on ND status (Table 2).

### 3.3. Multivariable Associations between Children’s BMI or Fat Percentage (FAT%) and ND Status

A logistic regression analysis was conducted containing children’s BMI status at pre-intervention as the dependent variable (overweight/obese versus underweight/healthy weight) and ND status (ND versus not having ND) as the independent variable, adjusting for parents’ BMI status (Table 3a). When analyzing the unadjusted results, the odds of children being in the overweight/obese category increased by a factor of 12.4 in the crude analysis when having an ND (95% CI: 2.53–68.26, *p* = 0.003) compared to those who did not have an ND. When adjusting for parents’ BMI status, the odds of children being in the overweight/obese category lowered to 9.08 (95% CI: 1.56–52.72, *p* = 0.014) when having an ND. 

A logistic regression analysis was also conducted containing children’s FAT% status at pre-intervention as the dependent variable (overfat/obese versus underfat/healthy) and ND status (ND versus not having ND) as the independent variable, adjusting for parents’ FAT% status (Table 3b). When analyzing the unadjusted results, the odds of children being in the overfat/obese category increased by a factor of 12.5 in the crude analysis when having an ND (95% CI: 3.04–66.70, *p* = 0.001) compared to those who did not have an ND. When adjusting for parents’ FAT% status, the odds of children being in the overweight/obese category remained significant, with an OR of 10.56 (95% CI: 2.27–49.20, *p* = 0.003) when having an ND.

### 3.4. Changes in Children’s BMI-SDS and Parents’ BMI and Fat Percentage (FAT%), Based on ND Status Comparing Changes between Time Points

**Figure 2:**
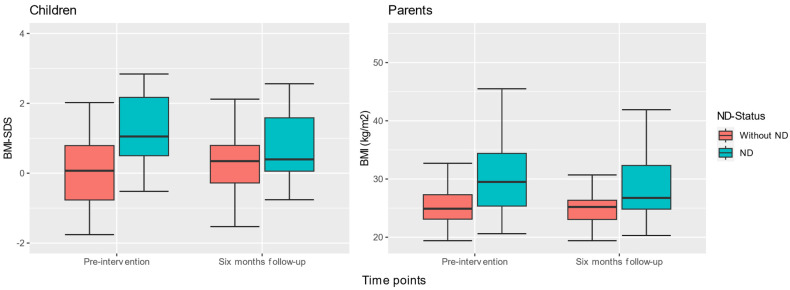
Distribution and changes in children’s BMI-SDS and parents’ BMI, based on ND status between baseline and six months follow-up.

When analyzing the distribution and changes in children’s BMI-SDS and parents’ BMI and FAT%, it was evident that children with an ND had significantly higher mean BMI-SDS (*M* = 0.9, *SD* = 1.27) at pre-intervention compared with children without an ND (*M* = −0.1, *SD* = 1.05) (*t*(17) = 6.041, *p* < 0.001) (Figure 2). The mean BMI-SDS lowered significantly between time points for children with an ND (*t*(17) = 4.408, *p* < 0.001), with no significant changes for children without an ND (*t*(32) = −1.893, *p* = 0.243). Similarly, parents of children with an ND had higher mean BMI (*M* = 29.9, *SD* = 6.38) at pre-intervention than parents whose children did not have an ND (*M* = 25.5, *SD* = 3.31). The BMI of parents whose children had an ND lowered significantly between time points (*t*(17) = 3.355, *p* = 0.003) but did not change significantly for parents whose children did not have an ND (*t*(32) = −1.189, *p* = 0.243). 

**Figure 3:**
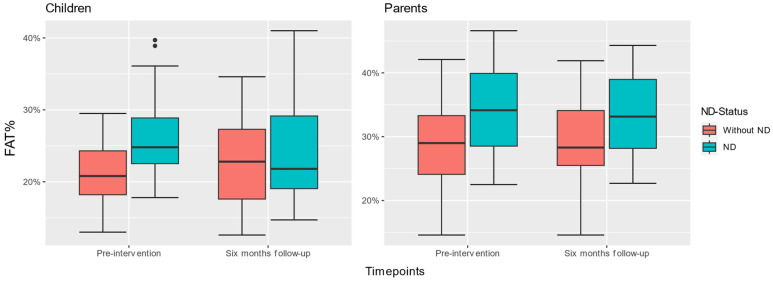
Distribution and changes in children’s and parents’ fat percentage (FAT%) between both time points, based on children’s ND status.

Figure 3 shows the distribution of children’s and parents’ FAT%. Children with an ND had significantly higher mean FAT% (*M* = 26.6, *SD* = 6.71) at pre-intervention compared with children without an ND (*M* = 21.2, *SD* = 4.39) (t(25) = −3.074, *p* = 0.005). The mean FAT% lowered significantly between time points for children with an ND (*t*(17), 2.646, *p* = 0.017) and increased significantly for children without an ND (*t*(32) = −2.223, *p* = 0.033). Similarly, parents of children with an ND had significantly higher mean FAT% (*M* = 34.3, *SD* = 7.07) compared with parents whose children did not have an ND (*M* = 28.7, *SD* = 7.01) (*t*(35) = −2.699, *p* = 0.011). The FAT% of parents whose children had an ND did not change significantly between time points (*t*(17), 0.702, *p* = 0.492) and did not lower significantly for parents whose children did not have an ND (*t*(32) = 0.041, *p* = 0.968). 

## 4. Discussion

This study aimed to analyze the body composition of parents and children, with and without an ND, participating in a food-based Taste Education intervention. To the best of our knowledge, body composition in a similar parent–child dyad sample has not been investigated previously or in other food-based interventions. Our study found that at pre-intervention children with an ND had significantly higher BMI, BMI-SDS, and FAT% measures than children without an ND. In addition, parents whose children had an ND were significantly younger and had higher BMI, fat mass, and FAT% than those whose children were without an ND. 

Although some studies have pointed to the protective aspect of fussy eating against over-eating due to a greater dislike of certain foods [23,32,115], we did not find evidence of this in our study [103], as only two children were identified as being underweight and underfat. Further, as one child had an ND and one did not, we were not able to find evidence of children with an ND being more likely to be underweight, as identified in some studies [23,24,25,26,27,28]. On the contrary, the odds of children being in the overweight/obese category versus underweight/healthy weight were raised over nine times when adjusting for parents’ BMI status. Similarly, the odds of children being in the overfat/obese category versus underfat/healthy were raised almost 11 times for children with an ND after adjusting for parents’ FAT% status. Unfortunately, studies are scarce on body composition in fussy-eating children. We could only identify one study where fussy-eating children had a lower fat-free mass at six years old than non-fussy eaters [26], which might point to a trajectory toward increased weight. Fussy eating may be a learned behavior, as parents’ adaptation to the food environment in which the children with fussy eating, with and without an ND, perhaps influences parents’ food choices. This has been noted in studies on maternal overweight and obesity and children’s control of food intake, where increased maternal weight was associated with children’s increased control over intake and food choices [11]. Further, parents may use food as a soothing mechanism for children with difficult temperaments [11] and as a reward or punishment system for children’s behavior, especially those who exhibit behavioral problems around mealtimes [14,31,116]. Studies have also shown that parents are more likely to use food as a reward if they perceive their child as a fussy eater, which may lead to selective eating behaviors and limited food choices [14,102,116,117].

Studies have indicated that parents with their own eating or weight concerns are more likely to control their child’s feeding environment and food intake [98,99], which may impact several mechanisms of weight and fussy eating. In our study, the relationships between children’s ND status and increased weight might have caused worries for parents about their children’s weight trajectory, or about their children heading towards obesity, and therefore sought out assistance in terms of our food intervention. 

It may also be helpful to compare our present results to a study on 104 Icelandic children with obesity (mean age; 12.0, *SD* = 3.5, mean BMI-SDS; 3.5, *SD* = 0.9), where almost half of the parents considered their child a fussy eater. In addition, nearly half of the children had a diagnosed disorder, including ASD with or without ADHD [20]. Approximately 8% of the children in our sample had obesity, which is slightly higher compared with recent estimates of 7% from The Development Centre for Primary Healthcare in Iceland (DCPHI) for overall obesity in children aged 6, 9, 12, and 14 years old [72]. However, of those children who had an ND, four had obesity (22.2%), and none of the 33 children that did not have an ND had obesity. Just under a third of the parents in our study had obesity, similar to obesity levels in Icelandic adults [75]. Therefore, the results raise concerns about the health status of Icelandic children with NDs. 

Children with an ND had significantly higher BMI-SDS and FAT% at pre-intervention than children without an ND. For the older children without an ND, there was a shift from healthy weight towards being overweight and increased FAT%, and as they may have been on the cusp of puberty, this might affect their weight status [105]. Although we did not record the amount of food that the children consumed in our intervention, we saw evidence of children eating a more varied diet and an indication of increased enjoyment of eating as measured by the Children’s Eating Behavior Questionnaire (CEBQ) [103], which might have influenced the older children who, based on age, have more autonomy regarding food choices. Similarly, parents whose children had an ND had significantly higher BMI and FAT% at pre-intervention than those with children without an ND. However, BMI-SDS and FAT% lowered significantly between pre-intervention and six months follow-up for children who had an ND while increasing significantly between time points for children without an ND. Parents of children with an ND participating in our study may have been worried about their children’s diets, as some already had obesity, motivating them more firmly to use the tools on fussy eating we provided in our intervention. There were no differences based on children’s ND status on the variety of fruit, vegetables, and other foods in our intervention [103], so it is unclear if the amount consumed was less. It is also unclear whether or which aspects of our Taste Education program affected the children’s weight status. However, studies have shown that improved eating behaviors may positively impact children’s weight status [14,28], possibly through parents’ reduced worry or stress [15,24,53,98,99]. In other papers recently published by the authors of the present study, the children from this sample decreased problematic mealtime behaviors [118], increased food variety, and decreased fussy eating [103] following our Taste Education intervention. Further, improved dietary variety, especially in terms of wholegrains, fruits, vegetables, and legumes emphasized in our Taste Education program, generally benefits children’s weight status, health, and overall well-being [18,24,25,40,97]. 

The strength and limitations of our recent Taste Education study have been detailed elsewhere [48,103,118]. The present study has many strengths. Primarily, the study is the first to investigate the body composition of a sample of fussy-eating children with and without an ND, and their parents, following a Taste Education intervention. Further strengths include objective body composition measurements using a Tanita Body Composition Analyzer—Tanita MC-780 [83] performed at two time points at six-month intervals and by the same team members with extensive experience. Some studies have found that in children, BIA may slightly overestimate change in FAT% with increased error in measurement with further changes in FAT% [119]. Thus, although the BIA technology is a considerably better measurement tool than relying on BMI alone, further examination of whether BIA can adequately measure changes in body composition over time is essential in larger samples of children from less homogenous groups.

The current study also had some limitations. The sample size was small as only 51 parent–child dyads were available for both measurements, with wide confidence intervals (CIs) for the multivariable regression analysis. Although there were no significant differences in background characteristics between those who received both measurements and those who did not, we could not rule out factors such as reluctance to participate, for example, due to weight concerns or increased weight between time points. There was no evidence of parents of children with an ND not wanting to attend both measurements, even though those children were more likely to be overweight or have obesity than TD children. Further, although care was taken to measure the participants at the same time of day at both time points, with an empty bladder, it was not possible to control the food or fluid intake right before the measurements. No comparison group was available for this study regarding children without fussy eating, and these data are unavailable in Iceland. Therefore, as the findings were temporal, no causal inference was possible as we were not able to isolate the effects of fussy eating on body composition. Although the sample of children represented a school classroom in Iceland well (i.e., a combination of children with and without an ND and being inclusive of other diagnoses), there was a very high proportion of well-educated parents in full-time work living in two-parent homes. The parents’ status indicates a high socioeconomic standing, so the results may not replicate lower socioeconomic circumstances; even more so, the high rate of obesity among participating children and parents is rather alarming. Finally, the increase in family-wise error across the reported statistical analyses was not controlled. Generally, we decided to report unadjusted alphas [113,114] as we consider this data analysis introductory, and not meant for intervention purposes, and we would like to emphasize replication with larger samples. 

## 5. Conclusions

The findings from this study underline the need for additional exploration into the relationships between children’s and parents’ body composition, and reasons for differences between BMI-SDS and FAT% of children with and without an ND, and similarly for the differences in BMI and FAT% between parents whose children had an ND and those who did not. Notably, there are no previously published studies on the associations between body compositions of fussy-eating children with and without an ND and their parents. The findings of the current study may be helpful as a step towards improving awareness of the weight status of children with an ND and fussy eating and possible associations between parents’ weight status. 

## Figures and Tables

**Table 1:** Characteristics of children and parents participating in the study, at pre-intervention (*n* = 51).

Child	
Children’s age in years, Mean (SD)	10.0 (1.44)
**Female**, *n* (%)	21 (41.2)
**Anthropomorphic measures**, Mean (SD)	
Height (cm)	143.0 (11.5)
Weight (kg)	37.3 (12.3)
BMI (kg/m^2^)	17.9 (3.5)
BMI-SDS	0.27 (1.21)
**Diagnoses**, *n* (%)	
Without ND	33 (64.7)
ND (ADHD, Autism, or both)	18 (35.3)
ADHD, primarily	4 (7.8)
ASD, primarily	4 (7.8)
Anxiety	10 (19.6)
Other	4 (7.8)
**Parent**	
Parents’ age in years, Mean (SD)	42.2 (4.94)
**Mother**, *n* (%)	42 (82.3)
**Education**, *n* (%)	
No higher education	3 (5.9)
Vocational education	11 (21.6)
University level	37 (72.5)
**Single parent household**, *n* (%)	5 (9.8)
**Occupational status**, *n* (%)	
Full-time occupation	34 (66.7)
Part-time occupation	6 (11.8)
Student	4 (7.8)
Other	7 (13.7)
**Anthropomorphic measures**, Mean (SD)	
Height (cm)	169.4 (7.9)
Weight (kg)	76.6 (16.9)
BMI (kg/m^2^)	26.9 (5.1)

Abbreviations: SD, standard deviation; BMI, body mass index; BMI-SDS, BMI standard deviation score; ND, neurodevelopmental disorder; ADHD, attention deficit/hyperactive disorder; ASD, autism spectrum disorder.

**Table 2:** Differences in body composition measurements of parents and children, based on children’s ND status (with or without an ND), at pre-intervention.

**Children’s body composition measurements, based on ND status**				
**ND Status**	Without ND (*n* = 33)	ND (*n* = 18)	*t*-test or *χ*^2^ (1)	*p*
**Measures**, Mean (SD) or *n* (%)				
Age in years	10.0 (1.55)	10.2 (1.25)	−0.492	0.624
Weight (kg)	34.8 (9.26)	41.9 (15.81)	−1.711	0.139
Height (cm)	141.2 (12.13)	146.2 (9.63)	−1.610	0.053
BMI (kg/m^2^)	17.2 (2.39)	19.2 (4.77)	−1.684	0.049
BMI-SDS	−0.1 (1.05)	0.9 (1.27)	−2.773	0.005
Overweight/obese	2 (6.1)	8 (44.4)	8.587	0.003
Fat mass (kg)	7.8 (3.34)	10.8 (8.06)	−1.508	0.067
FAT%	21.2 (4.39)	26.6 (6.71)	−3.074	0.001
Overfat/obese	3 (9.1)	10 (55.5)	10.906	0.001
FFM (kg)	27.0 (6.44)	30.9 (8.37)	−1.742	0.066
Bone mass (kg) *	1.4 (0.32)	1.7 (0.39)	−2.156	0.026
**Parents’ body composition measurements, based on children’s ND status**				
**ND Status**	Without ND (*n* = 33)	ND (*n* = 18)	*t*-test or *χ*^2^ (1)	*p*
**Measures**, Mean (SD) or *n* (%)				
Age in years	43.6 (4.97)	39.6 (3.75)	3.278	0.004
Weight (kg)	72.6 (13.60)	84.0 (20.21)	−2.137	0.021
Height (cm)	169.1 (7.83)	170.1 (8.21)	−0.435	0.661
BMI (kg/m^2^)	25.5 (3.31)	29.9 (6.38)	−2.893	0.001
Overweight/obese	15 (45.5)	14 (77.8)	3.774	0.045
Fat mass (kg)	21.0 (7.28)	29.4 (11.01)	−2.916	0.002
FAT%	28.7 (7.01)	34.3 (7.07)	−2.700	0.009
Overfat/obese	12 (36.4)	12 (66.7)	3.163	0.046
FFM (kg)	51.6 (10.62)	55.1 (11.55)	−1.065	0.280
Bone mass (kg)	2.6 (0.50)	2.8 (0.54)	−1.100	0.266

Abbreviations: SD, standard deviation; BMI, body mass index; BMI-SDS, BMI standard deviation score; FAT%, fat percentage; FFM, fat-free mass. All *p*-values from t-test, χ^2^ test or Fisher’s exact test. Unadjusted alphas are reported. * Boys had significantly higher bone mass than girls.

**Table 3:** (**a**) Multivariable associations between children’s BMI status at pre-intervention; overweight/obese category versus underweight/healthy weight, and ND status (with or without ND), using binary logistic regression analysis (unadjusted and adjusted analysis), *n* = 51. (**b**) Multivariable associations between children’s fat percentage (FAT%) status at pre-intervention; overfat/obese category, versus underfat/healthy, and ND status (with or without ND), using binary logistic regression analysis (unadjusted and adjusted analysis), *n* = 51.

Multivariable Associations Using Binary Logistic Regression							
(**a**)							
	*n*	*β*	Unadjusted OR (95% CI)	*p*	*β*	Adjusted OR (95% CI)	*p*
Parents’ BMI category	51						
Underweight/healthy vs	22						
Overweight/Obese	29				2.24	9.45 (1.10–81.49)	0.040
ND status	51						
Without ND vs	33						
ND	18	2.52	12.4 (2.53–68.26)	0.003	2.21	9.08 (1.56–52.72)	0.014
(**b**)							
	*n*	*β*	Unadjusted OR (95% CI)	*p*	*β*	Adjusted OR (95% CI)	*p*
Parents’ FAT% category	51						
Underfat/healthy vs	27						
Overfat/Obese	24				0.91	2.12 (0.46–81.50)	0.334
ND status	51						
Without ND vs	33						
ND	18	2.53	12.5 (3.04–66.70)	0.001	2.36	10.56 (2.27–49.20)	0.003

Abbreviations: BMI, body mass index; ND, neurodevelopmental disorders; CI, confidence intervals; OR, odds ratio.

## Data Availability

The datasets generated during and/or analyzed during the current study are available from the corresponding author upon reasonable request.
